# Dogs’ Detection of Symptomatic and Asymptomatic SARS-CoV-2 by Non-Working Dogs: Feasibility and Limits Under Controlled Laboratory Conditions

**DOI:** 10.3390/ani16030480

**Published:** 2026-02-04

**Authors:** Jennifer Cattet, Frédérique Retornaz, Florine Munier, Catherine Collignon, Florence Gaunet

**Affiliations:** 1 Center for the Study of Medical Alert Canines (CMAC), and Medical Mutts Service Dogs, 6120 Allisonville Rd, Indianapolis, IN 46220, USA; jen@medicalmutts.com; 2 Unité de Soins et de Recherche en Médecine Interne et Maladies Infectieuses, Hôpital Européen, 6 Rue Désirée Clary, 13003 Marseille, France; 3 Chiens d’Assistance pour Diabétiques Centre Domazane (ACADIA), 295 Chemin des Buis, 26250 Livron sur Drôme, France; 4 Animalin Boutique, 173 Rue des Jardins, 32450 Aurimont, France; 5 Centre de Recherche en Psychologie et Neurosciences (UMR 7077), Aix-Marseille University and CNRS, 3, Place Victor Hugo, 13003 Marseille, France

**Keywords:** scent detection, dogs, volatile organic compounds, asymptomatic, symptomatic, COVID-19

## Abstract

Dogs can detect volatile organic compounds (VOCs) associated with infectious diseases, but their ability to distinguish between symptomatic and asymptomatic SARS-CoV-2 infections has not been fully established. In this exploratory proof-of-concept study with a limited number of dogs, eight non-working dogs (without experience in scent detection) began training with either symptomatic or asymptomatic Delta samples; seven reached the testing phase and were then evaluated on the alternate group to assess cross-generalization. Training initially used a yes/no protocol but was adapted to a line-up design after poor performance. When presented with novel Delta samples from the other category, dogs significantly discriminated them from controls, achieving a mean sensitivity of 71% and a specificity of 51%, with no difference between training groups. In contrast, performance dropped when dogs were tested with a small number of asymptomatic Omicron-positive samples from vaccinated individuals, with a mean sensitivity of 35% and a specificity of 55%. Dogs can thus generalize across symptomatic and asymptomatic COVID-19 cases for the Delta variant; performance decreased for Omicron samples, likely due to altered VOC profiles in vaccinated individuals. While proof-of-concept feasibility was demonstrated with a line-up protocol for detecting asymptomatic scent, detection dogs should not currently be recommended for large-scale screening; the findings underscore the need for standardized protocols and variant-specific retraining.

## Simple Summary

Dogs can detect odors associated with infectious diseases, but their ability to distinguish between symptomatic and asymptomatic SARS-CoV-2 infections has not been fully established. In this exploratory proof-of-concept study with a limited number of dogs, eight non-working dogs began training with either symptomatic or asymptomatic samples of the Delta variant. We next tested whether dogs trained with asymptomatic samples could detect symptomatic samples, and vice versa. Poor performance in a task where odors had to be strictly identified (yes/no protocol) led us to use a simpler task in which dogs had to discriminate the targeted odor among five other samples. When presented with novel Delta samples, each group of dogs significantly discriminated them from controls, and the two groups performed equally well. In contrast, performance dropped when dogs were tested with a small number of asymptomatic Omicron-positive samples from vaccinated individuals. These results show that dogs can generalize across symptomatic and asymptomatic COVID-19 cases for the Delta variant but showed reduced performance with Omicron samples, likely due to altered odor profiles in vaccinated individuals. While proof-of-concept feasibility was demonstrated in a small group of dogs using a categorization task for detecting asymptomatic scents, detection dogs should not currently be recommended for large-scale screening.

## 1. Introduction

Several diseases are known to produce specific scents in patients, which are excreted as volatile organic compounds (VOCs) and can be detected within seconds by dogs. Medical detection dogs show great potential for use as alternative diagnostic tools not only for organic diseases [1], such as epileptic or diabetic crises [2,3] or cancer [4], but also infectious diseases [5,6].

The detection of scents produced by infections involves distinct challenges compared to those associated with organic diseases. Variables include differences in viral load, the symptomatic state of individuals (ranging from symptomatic to pre-symptomatic and asymptomatic), the context in which the dog is working, and the evolution of viral variants over time. The high mutation rates of viruses, coupled with short generation times and large population sizes, allow viruses to rapidly adapt to the host environment, which generates new variants over time. Another underlying issue is whether the detectable scent originates from the virus’s metabolism itself or from the abnormal functioning of affected organs (lungs, liver, sinuses, intestines, etc.); this issue is unclear at the moment, remaining unresolved [7]. Additionally, organ states and medication use can vary widely between persons based on their metabolic conditions, further increasing the variability of scent profiles. This variability poses a significant challenge for dogs tasked with identifying virus-positive individuals. Achieving consistent performance under such variable conditions likely depends on dogs reaching a sufficient and standardized level of training [8].

During the 2020–2023 SARS-CoV-2 pandemic, particularly due to pre- or asymptomatic transmission, disease control remained challenging [9]. For COVID-19 detection, dogs have been used to complement the RT-PCR (reverse transcription polymerase chain reaction) conventional detection method. In the systematic review by Meller et al. [10], the use of dogs’ olfaction as a reliable COVID-19 screening tool was evaluated. Twenty-seven studies from thirteen countries were used as material for two independent study quality assessment procedures. Potential confounding factors, such as study design, patient/sample selection, dog characteristics, training protocols, and sample types/treatment, were considered. Only four and six studies, respectively, had a low risk of bias and were of high quality. Furthermore, these ten studies indicated that dogs distinguished between COVID-19-positive and -negative persons: while the four QUADAS-2 (Quality Assessment of Diagnostic Accuracy Studies, version 2) non-biased studies revealed sensitivity and specificity ranging from 81–97% to 91–100%, the six high-quality studies, according to the general evaluation system, resulted in sensitivity and specificity ranging from 82–97% to 83–100%, respectively. The other studies presented methodological, quality, and bias concerns. Notably, the variants or the presence of symptoms in COVID-19-positive persons were not specifically addressed as factors by Meller et al. Given the small number of papers that remained sufficiently reliable to be analyzed, these authors concluded that, as for canine explosive detection, standardization and certification procedures should be established for medical scent detection dogs.

A major challenge for controlling the spread of a virus in the population is the detection of symptomatic (S) or asymptomatic (AS) patients against controls (C). To our knowledge, no study has specifically examined whether dogs can discriminate between these groups in the case of COVID-19.

Research in related areas provides useful insights. Three studies have investigated scent detection by dogs and electronic noses (eNose) in other infectious diseases. Guest et al. [11] showed that trained dogs identified asymptomatic malaria-infected individuals by their scent, suggesting a role for canine detection at borders or in malaria-elimination regions. In a later study, Guest et al. [12] trained dogs to recognize the scent of asymptomatic SARS-CoV-2 cases (patients not requiring hospitalization, able to walk short distances, and stand for up to 15 min). Both dogs and organic semiconductor sensors (OSCs) detected asymptomatic and mild cases with a high degree of accuracy under laboratory conditions. However, they did not compare asymptomatic with symptomatic patients directly, leaving unresolved whether dogs could generalize across these groups, which is the central aim of our study. Similarly, Grandjean et al. [13] reported that dogs achieved a higher sensitivity than nasopharyngeal antigen testing, though with lower specificity. While this highlights the diagnostic potential of canine scent detection, the study did not assess whether detection varied by symptom status, which we addressed explicitly in the present work.

SARS-CoV-2 variants arise through the accumulation of mutations that can alter viral replication dynamics and host immune responses. Such changes may influence host metabolism and inflammatory processes involved in disease expression. Emerging evidence suggests that canine detection performance has varied with the emergence of new SARS-CoV-2 variants, including reduced performance for Omicron compared with Delta, supporting the hypothesis that variant-related biological differences may affect odor profiles [10].

Together, these findings demonstrate that trained dogs can detect asymptomatic infection under controlled conditions. However, it remains unclear whether dogs can generalize between symptomatic and asymptomatic patients, a question that previous studies did not directly address and which forms the central focus of our investigation.

In another study, Bax et al. [14] used an electronic nose (eNose) to analyze exhaled breath from 33 SARS-CoV-2-infected patients, 25 suffering from respiratory failure and 8 asymptomatic, as well as 22 control subjects. The features identified by the Boruta algorithm were significantly different in SARS-CoV-2 patients with respiratory failure compared with both controls and asymptomatic SARS-CoV-2 patients. This finding indicates that VOC signatures may vary depending on symptom severity and clinical status. It underscores the need to test whether detection dogs face similar challenges when distinguishing between symptomatic and asymptomatic cases, a key focus of our study.

Ungar et al. [15] trained two dogs with heterogeneous samples, including Alpha from hospitalized patients and Alpha, Delta, and Omicron from known COVID-19-positive individuals. During testing, these dogs achieved high agreement rates (95–96% positive, 94–96% negative) and remained accurate even with Omicron subvariants, suggesting that VOC profiles may be stable across variants. Interestingly, the dogs also alerted to an asymptomatic relative who later tested PCR-positive, highlighting their potential to detect infection before clinical confirmation. In contrast, Ozgur et al. [16] reported inconsistent VOC signatures across SARS-CoV-2 variants, indicating that olfactory detection may not always generalize across viral evolution. VOC might indeed differ according to the smell produced by a different set of affected organs or systems; there is no reason to think that the variant has a specific odor.

There is increasing interest in utilizing trained dogs as alternative methods for screening asymptomatic individuals for infectious diseases [17]. However, detection accuracy has been shown to vary depending on the virus and study design. This variability reinforces the importance of developing standardized protocols for canine detection. In our study, we address this issue directly by discussing the yes/no and line-up procedures under controlled conditions.

A final issue concerns the training protocols used for canine scent detection. Two main approaches exist: line-up protocols, which assess relative discrimination among multiple samples, and yes/no procedures, which test absolute recognition of an odor signature. Each has strengths and limitations; here, we evaluated both under controlled conditions [8,18].

This study aimed to determine whether dogs trained on symptomatic COVID-19 patients could detect asymptomatic cases, and vice versa, and to test whether such generalization was preserved across viral variants (Delta vs. Omicron). To address this, we used two detection protocols (yes/no vs. line-up) under controlled, double-blind conditions. Because of infection control challenges in early 2021 [16], we initially adopted the yes/no procedure, which is considered a conservative method, before later transitioning to the line-up protocol. This design allowed us to assess the strengths and weaknesses of both approaches and to directly evaluate whether symptomatic and asymptomatic SARS-CoV-2 infections produce discriminable odor signatures, and whether detection performance was maintained across two different variants.

Given all confounding factors mentioned earlier, among them the distinguishability of asymptomatic vs. symptomatic COVID-19 scent, we questioned whether a distinguishable scent exists for symptomatic and asymptomatic persons. We therefore designed a proof-of-concept study to test a protocol not previously applied in canine scent detection: specifically, whether dogs trained on samples from symptomatic (S) SARS-CoV-2-infected patients could detect samples from asymptomatic (AS) SARS-CoV-2-infected patients, and conversely. This question had not been examined in earlier studies. Given the infection control challenges in January 2021 [16] and because line-up paradigms limit the computation of specificity and reliability, we first adopted a conservative training and testing procedure using a Yes/No paradigm (also known as a Go/No Go paradigm).

Given the absence of prior studies directly comparing canine detection of symptomatic versus asymptomatic SARS-CoV-2 infections, the study was designed as exploratory with respect to symptom status for the Delta variant, and no specific hypothesis was formulated for this comparison. However, based on emerging evidence suggesting that SARS-CoV-2 variants may differ in biological characteristics relevant to odor production, we hypothesized that performance might decline with newer viral variants such as Omicron.

## 2. Materials and Methods

To address these aims, we conducted a randomized, double-blind, prospective study comparing the performance of non-working dogs (with no prior experience in scent detection or operational detection tasks) trained to detect symptomatic versus asymptomatic COVID-19 cases, first using a yes/no protocol and later a line-up procedure. The study was approved by the French institutional review board for human studies (CPP SUD-EST I) and conducted in accordance with the Declaration of Helsinki, Good Clinical Practices, and local ethical and legal requirements (trial registration: ClinicalTrials.gov NCT04970563). Informed consent was obtained from all patients prior to sample collection. This study was conducted in accordance with the Declaration of Helsinki and its latest revision in 2013, the legal requirements of France, and the institutional guidelines of Aix-Marseille University. The Animal Ethics Committee n° 71 of Marseille, France, additionally reviewed the project and confirmed that no authorization was required, as the protocol did not affect dog welfare.

### 2.1. Material

#### 2.1.1. Human Participants’ Scent Sampling

##### Subjects

A total of 160 human subjects (≥18 years old) were included in the study by Hôpital Européen in Marseille. All participants were screened for COVID-19 using RT-qPCR with a nasopharyngeal swab (Delta) from June 2021 to January 2022. RT-qPCR testing was performed under routine conditions by using the UltraGene Combo2Screen SARS-CoV-2 Assay (ref 139b) (ABL SA Group, Luxembourg) and Chemagic™ viral DNA/RNA 300 kit H96 (ref. CMG-1033-S) on a Chemagic™ 360-D instrument (PerkinElmer, Inc., Austin, TX, USA) according to the manufacturer’s instructions [PerkinElmer. Instructions for the Perkin Elmer new coronavirus nucleic acid detection kit. V6.0. 2020].

AS patients (*n* = 50) presented positive RT-PCR for SARS-CoV-2 without any symptoms (fever, cough, anosmia, diarrhea). S patients (*n* = 50) presented positive RT-PCR for SARS-CoV-2 with at least one symptom (fever, cough, anosmia, diarrhea). Healthy persons/controls (*n* = 60) presented negative RT-PCR for SARS-CoV-2 and no symptoms (fever, cough, anosmia, diarrhea) at Day 0 and Day 7.

All participants were recruited after the RT-PCR test results were sent from the European Hospital laboratory to the principal investigator (FR). To increase sample diversity, participants were included from different populations: inpatients, outpatients, at-home patients, physicians, nurses, and staff members from the hospital.

Demographic data (age, sex, body mass index), symptomatic status (for symptomatic patients: presence of fever, cough, anosmia, diarrhea), comorbidities and risk factors (tobacco use, diabetes, hypertension, dyslipidemia, cardiovascular disease, active cancer, severe renal failure), previous COVID-19 infection and vaccinal status (0 to 3 doses and type of vaccines when available) were recorded.

Inclusion criteria were as follows:

- 18 years of age or older;
- Free, informed, and express consent;
- Affiliation with or coverage by a social security plan;
- Patients infected with SARS-CoV-2 with positive RT-PCR on nasopharyngeal or salivary swabs and no symptoms (fever, cough, anosmia) were included in the asymptomatic group. *n* = 50;
- Patients infected with SARS-CoV-2 with positive RT-PCR on nasopharyngeal or salivary swabs and at least one symptom (fever, cough, anosmia, and/or diarrhea) were included in the symptomatic group. *n* = 50;
- Participants not infected with SARS-CoV-2 with negative RT-PCR on nasopharyngeal or salivary swabs and no symptoms (fever, cough, anosmia, diarrhea) at Day 0 and Day 7 were included in the Healthy control group. *n* = 60.

Non-inclusion and exclusion criteria for creating scent samples included the following:

- Persons under legal protection measures or the safeguard of justice;
- Life expectancy of less than 3 months.

Healthy controls with positive RT-PCR tests at Day 7 were excluded from the control.

- If asymptomatic, samples (body sweat compresses and masks) were collected, and participants were integrated into the asymptomatic group.
- If symptomatic, they were excluded from the study, and their samples were destroyed.

##### Odor’s Samples

Once participants were assigned to one of the three groups, samples were collected either at Hôpital European Marseille or at the participant’s home. Sweat samples and breath odor were self-collected by patients under the supervision of the healthcare staff (physician) who instructed them on the procedure.

To avoid contamination, the physician wore two pairs of new gloves, FFP2 masks, and full COVID-19 safety protective equipment when collecting all samples (regardless of the COVID-19 status). A new set of protective equipment was used for each participant. For each participant, six consecutive identical samples were collected.

For the sample collection procedure, participants were asked to wear a surgical mask for one minute, and a 2 × 2-inch gauze swab was rubbed on the inguinal area and then kept in contact with the skin for one minute. Both mask and gauze swabs were inserted in a hermetic safety bag (95 KpA) by the participants. Sweat and breath samples were intentionally combined and treated as a single odor source; no attempt was made in this study to compare detection accuracy between sweat and breath samples. The safety bag was placed in two additional insulating bags (triple packaging). The procedure was repeated six times for each participant, producing six series of samples per participant. We assumed equivalent volatile compound concentration across repeated samples.

##### Storage and Mailing Procedure

The samples were next stored for at least 14 days in a dedicated freezer (−20 °C) at the European Hospital laboratory to limit bacterial growth and reduce viral viability on the masks and sweat samples. All samples were stored in the same way, whether they were Omicron or Delta. However, prior research has shown that SARS-CoV-2 can remain stable and potentially infectious at freezing temperatures (−20 °C) for extended periods [18]. Therefore, subsequent storage at ultra-low temperatures (−80 °C) was used to maximize biosafety and sample integrity.

Samples collected were divided (see below), sent, and stored by the detection dog teams (ACADIA and ANIMALIN) in a dedicated refrigerator until use (i.e., within two weeks of receipt) in a secure room, first for the training phase, and later for the test phase only (ACADIA school only).

#### 2.1.2. Dog Participants

Eight dogs were recruited for this study, aged between 1.5 and 9 (mean = 4.06, SD = 3.05), including six males and two females. All were neutered.

Two dog training organizations each provided four of the dogs participating in the study: ACADIA, a not-for-profit organization dedicated to the training of service dogs as diabetes alert dogs, and Animalin, a pet dog training school utilizing scent training in sports and in behavior modification protocols. All the dogs lived with their respective owners and came to the training center 2 to 4 times per week over a four-month period of training and preparation. The dogs were of a variety of breeds and breed mixes and came from diverse backgrounds, most from shelters and rescues (see Table 1). They were selected for their high sociability, their ability to adapt to novel situations, and their good health. They had received prior basic obedience training but had no prior experience in scent detection. Dogs were not selected based on specific suitability for scent discrimination.

### 2.2. Experimental Setup for the Testing Phase and Behavioral Analysis

**Figure 1:**
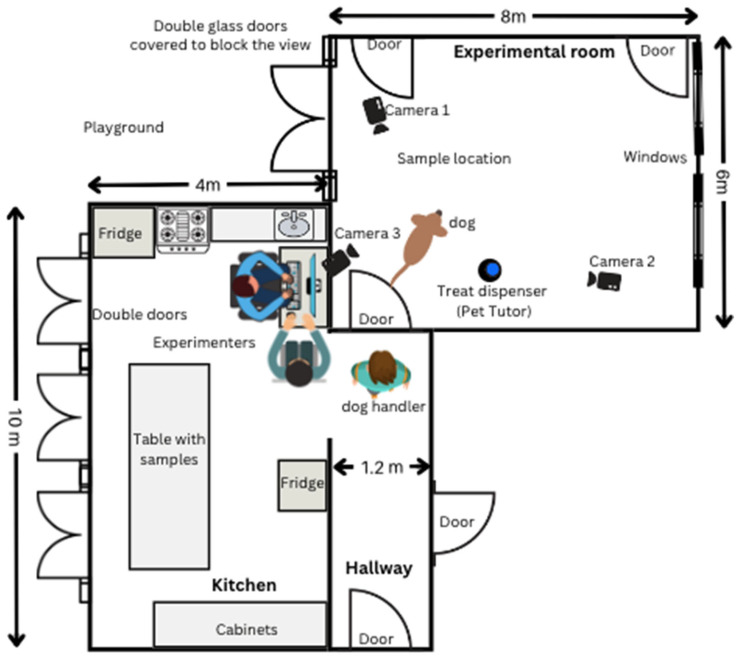
Experimental setup.

The testing phase was conducted in a spacious room at the ACADIA headquarters (8 m × 6 m) (see Figure 1). Two cameras mounted on tripods were strategically positioned within the room to record the dogs’ behavior (Cameras 1 & 2). A third camera (Camera 3), connected directly to a computer, allowed for real-time remote monitoring.

A remote treat dispenser (Pet Tutor) was placed inside the testing room to deliver rewards, which were triggered by an experimenter stationed in an adjacent control room. This setup ensured that reinforcement could be provided promptly while minimizing the experimenter’s influence on the dog’s behavior.

All dogs’ responses were identifiable in real time by the experimenter during the testing sessions and were later reviewed offline by the judge, who recorded each instance of correct and incorrect responses [19].

#### 2.2.1. Scent Box Training—Yes/No Procedure

The choice of the yes/no procedure was supported by the work of Gadbois and Reeve [20]. The authors argue that multiple-alternative forced-choice tasks, such as line-up procedures with several choices, may misrepresent the accuracy of canines, as they rely on relative discrimination between stimuli. Instead, detection tasks like yes/no procedures provide a more robust and reliable measure of sensitivity in sensory detection and diagnosis since they indicate absolute identification/recognition of a stimulus. Indeed, the authors emphasize that the yes/no procedure allows for a solid assessment of performance and the ability to identify the specific type of errors made by the dogs, such as false alarms or misses. By utilizing this approach, the authors argue that the yes/no procedure enables a more accurate assessment of the dog’s detectability or discriminability.

The dogs learned through positive reinforcement to show a final trained response: “stand-stare” at the target odor. This method was based on classical and operant conditioning, as developed by B.F. Skinner [21] and E. Thorndike [22]. Through successive approximations, the trainers marked any correct behavior with a clicker (a distinct noise maker) and gradually shaped the behavior until the dogs reliably stood and stared at the target odor for at least 3 consecutive seconds (final indication behavior). Incorrect responses received no reinforcement and were ignored. This behavior was trained as the dog signal for identifying SARS-CoV-2. A second behavior was taught for “Normal” samples: sitting. The dogs were presented with one sample at a time and trained to provide the corresponding behavior: “stand-stare” at a SARS-CoV-2 sample (AS and S) and “sit” in front of a Normal (N) sample.

The eight dogs were divided into two distinct training groups. The first group consisted of four dogs, trained exclusively with samples obtained from symptomatic (S) and healthy patients (N). The second group also comprised four dogs, trained solely with samples from asymptomatic (AS) and healthy patients (N). Furthermore, the dogs were evenly distributed between two training schools, ACADIA and ANIMALIN, with two dogs from each group being trained at each school. Specifically, two dogs from the asymptomatic group were trained at each school, along with two dogs from the symptomatic group. Note that one dog, Icare, from ANIMALIN school, was included in the pool of dogs three months later than the first three dogs, receiving only one month of training, while the seven others received four months of training. The training regimen involved exposing each dog to 20 normal (negative) SARS-CoV-2 samples that were from the same control persons for both groups of dogs and 10 SARS-CoV-2 (positive) samples of either symptomatic or asymptomatic Delta strains that were, by definition, from different patients, to develop their detection capabilities (see Table 2).

For both dog pools, the same human participants’ scents were used; however, each dog was exposed only to samples from its assigned category (symptomatic or asymptomatic), ensuring that differences in performance reflected symptom status rather than individual-specific odor characteristics. Characteristics of these participants are described in the Supplemental File, Part I.

For the training, samples were placed in 1 oz aluminum containers with holes in the lid, which were then placed inside larger containers (8 oz) also with lid holes. This setup ensured that direct contact between the dogs’ noses and the samples was not possible. During training, a single container holding either a Normal or SARS-CoV-2 sample was placed on a plywood box measuring 30 cm in height, 20 cm in width, and 20 cm in depth. To ensure stability during investigation, a hole was cut at the top of the box, matching the diameter of the 8 oz container, so the container could be securely inserted without moving. Only one stimulus was presented at a time, and the dogs entered and then left the room after each training trial. They were trained to approach the box, sniff the container, and exhibit either a hold-stare response if the sample was a SARS-CoV-2 sample (“Yes”) or a sit response if the sample was normal (“No”). To minimize human influence, the rewards were delivered through a remote-controlled treat dispenser (Pet Tutor). This allowed the trainer to remain outside the training room, eliminating any direct interaction. Training was considered successful when the dogs consistently displayed the appropriate behavior at least 80% of the time.

Although there were initial signs of progress and some dogs appeared to grasp the task, their performance remained inconsistent throughout the four months of training (one month for Icare), with fluctuations and unpredictable outcomes. The dogs struggled to provide consistent responses and faced difficulties in generalizing from the samples used during training. Achieving a consistent 80% accuracy proved to be challenging for most of the dogs.

All dogs were gathered at ACADIA to complete the learning phase. To familiarize the dogs with the experimental testing room, they were allowed ~5 min. minutes to explore it. Next, for a final training session, dogs were presented with the same 20 stimuli from the same participants used previously, but the third series of samples was used because series 1 and 2 of samples had been used extensively. We had three 3 replicates from each person. Dogs showed inconsistent performance: while they displayed the correct behavior for some samples, in other cases, they appeared to respond randomly, and in some instances seemed to have memorized specific behaviors for specific samples. Overall, they were unable to achieve the required 80% performance criterion. The success rate ranged from 40% to 70%, falling below the desired learning threshold.

In light of these challenges, we decided to alter our training strategy and reintroduce the presentation of samples in a line format. While acknowledging that the line presentation may prompt the dogs to seek out salient odors rather than specific ones, and that recognition in this case is not absolute, it was considered appropriate to investigate the scientific questions above using both procedures and to discuss their respective strengths and limitations.

#### 2.2.2. Discrimination Training—Line-Up Procedure

##### Material: Room and Experimental Setup

The same rooms and experimental setup were used in the previous protocol (see Figure 1). The line-up was placed in the same location as the single sample in the yes/no procedure. The video footage captured by the cameras was transmitted to a computer located in an adjacent room for observation and analysis.

One board (160 cm long × 13 cm wide × 2 cm thick) with six holes, each the same diameter as the sample containers (8 cm), was used for the line-ups. The samples were fitted tightly into the holes to ensure stability and avoid any shifting caused by the dogs’ interaction. Six samples were evenly distributed: one SARS-CoV-2 (S or AS of Delta strain) sample and five normal (N) samples. The board was elevated at the same height as in the Yes/No setup and was placed on two boxes (30 cm × 20 cm × 20 cm), one at each end.

##### Procedure

Training

All dogs underwent a two-day re-training session with the samples presented in a line-up.

Each dog was individually introduced into the test room with no human present, and the door was securely closed behind them.

Each dog continued to be trained with either the AS or S samples from the third series of samples from the same participants they had previously been trained with; they were never exposed to the other type of COVID-19 samples. However, the Normal (N) samples were new to the dogs and also from the third series.

The samples were randomly selected and presented in sets of one COVID-19 sample (AS or S) and five Normal samples (see Supplemental file Part II for participant characteristics). Inside the room, dogs were free to explore the six-sample setup and sniff the containers. Once a dog located the target sample and exhibited the indication behavior (holding the nose over the sample—hold-stare), they were promptly rewarded using the Pet Tutor. The dog was then called out of the room. If the sample was not found within one minute, the dog was called out without a reward. If a dog performed the indication behavior (hold-stare) on a Normal sample, this response was scored as incorrect, and no reward was delivered; the trial was then ended and the dog was called out of the room. All containers and line-up supports were cleaned between dogs using disinfecting wipes and water, and samples were handled exclusively with gloves.

This procedure was repeated until all dogs reached the learning criterion of at least 80% success. Most dogs were able to reach an 80% success rate during two consecutive days of training. Only Icare did not meet the minimum required.

Baseline

On day three, the seven dogs were assessed in a baseline phase with the same samples they had been trained with, comprised of five trials. A total of five COVID-19 samples (S or AS) and ten Normal samples were randomly selected and presented during this phase. The dogs were considered ready for testing since they successfully located the COVID-19 sample while ignoring the Normal samples, at least 80% of the time. The dogs were rewarded with the remote treat dispenser when finding the target samples.

For the baseline test, each dog completed five line-ups, each presenting six samples (five N and one AS or S), with the same set of 15 samples presented at random. This baseline phase intentionally used the same samples as those presented during training, in order to confirm that dogs had successfully learned the discrimination task under stable conditions. This phase was not intended to assess generalization, but rather to establish that each dog could reliably perform the trained task prior to testing with novel samples. In contrast to the baseline phase, all samples (COVID and Normal) used during the test phase (Delta and Omicron) were entirely novel and had not been presented either during training or baseline.

Each dog continued to be presented with either the AS or S samples from the third series of samples from the same participants they were previously trained with; again, they were never exposed to the other type of COVID-19 samples.

Test of samples

During the study, Omicron samples became available due to the evolution of the epidemic at the time of sample collection. The test phase was therefore divided into two parts.

Part 1 Delta: Each dog participated in ten trials, with each trial consisting of a line-up of six samples. Across all trials, 5 S samples, 5 AS samples, and 25 Normal samples were used. Each line-up contained one COVID-19 (S or AS) sample and five Normal samples randomly selected and presented (see Supplemental file Part III for participant characteristics). The same samples were used for all dogs. In this test, dogs were presented for the first time with samples from the category they had not previously trained on (either S or AS).

Part 2 Omicron as a pilot study (next day): Without any additional training, the dogs underwent five test trials similar to those for the Delta strain. Each trial included one Omicron AS sample and five Normal samples. The Omicron samples came from five different asymptomatic patients (see Supplemental file Part IV for participant characteristics). The Normal samples were randomly selected from the 25 Normal samples used in Part 1 (Delta). The samples were randomized for each dog. Normal samples for the Omicron test were drawn from the same pool of 25 Normal samples used in the Delta test phase. However, line-ups were newly randomized for each dog and each trial, such that not all Normal samples had necessarily been encountered previously: some were presented once, others multiple times, and some not at all. In contrast, all Omicron-positive samples were novel.

All COVID-19 samples used in both tests were completely novel to the dogs, ensuring that they encountered unfamiliar scents compared with the learning and baseline phases, as well as throughout the trials. Within the test phase, a finite set of Normal samples was reused across trials to construct multiple line-ups while maintaining blinding and feasibility, given the finite sample set; however, each specific trial (i.e., each line-up configuration) was presented only once to each dog. Because dogs completed multiple trials and encountered some of the same Normal samples, observations are not fully independent. Analyses, therefore, reflect trial-level performance and should be interpreted cautiously. Accordingly, inferential statistics should be viewed as a trial-level comparison rather than independent subject-level estimates.

##### Behavioral Collection and Statistical Analysis for the Line-Up Protocol

We first define the type of response we collected from dog behaviors using the videos.

- TP: True Positive. A scent sample from a COVID-19 person is indicated by the dog as a scent sample from a person with COVID-19.
- FP: False Positive. A scent sample from a healthy person is indicated by the dog as a scent sample from a person with COVID-19.
- TN: True Negative. A scent sample from a healthy person is indicated by the dog as a scent sample from a healthy person.
- FN: False Negative. A scent sample from a COVID-19 person is indicated by the dog as a scent sample from a healthy person.

If the dog failed to produce the trained indication on a COVID-19-positive sample, the outcome was scored as a false negative. If the dog produced an indication on a Normal sample, the outcome was scored as a false positive. Thus, all trials were classified unambiguously as true positive, true negative, false positive, or false negative.

The Friedman test was used to test the following hypotheses (with alpha = 0.05 and Bonferroni adjustments):

- True positive responses are above chance.
- True negative responses are above chance.
- The rate of correct responses for the four dogs trained with asymptomatic COVID-19 odor samples does not differ significantly from that of the four dogs trained with symptomatic COVID-19 odor samples.

Sensitivity and specificity were calculated per sample. In addition, sensitivity and specificity were calculated and presented below, combining the responses of the eight dogs. Finally, sensitivity and specificity were calculated separately for the two groups of four dogs. A 95% confidence interval was used.

Sensitivity = TP#/(TP# + FN#)

Specificity = TN#/(TN# + FP#)

## 3. Results

One out of seven dogs (i.e., Icare) did not reach the baseline learning criterion of 80% correct responses; he was therefore excluded from subsequent analysis. In each trial, only one out of the six cans contained the COVID-19 odor; hence, the chance sensitivity (the proportion of actual “positives” correctly identified) was 16.5%.

### 3.1. Delta Samples—Part One

Sensitivity ranged from 50% to 90% with an average of 71.4% (95% CI: 57.7–82.8%), significantly greater than chance: *p* < 0.0001 (Chi-squared). Specificity had an average of 50.7% (95% CI: 45.5–56.0%).

Three out of seven dogs reached 80% sensitivity, while four had lower performances (see Table 3).

### 3.2. Omicron Samples—Part Two (Pilot Study)

Sensitivity ranged from 0% to 80% with an average of 54.9% (95% CI: 47.5–62.2%), showing a trend towards significance (*p* = 0.0585, Chi-square; Table 4). Specificity had an average of 54.9% (95% CI: 47.5–62.2%). Only one dog reached 80% sensitivity.

### 3.3. Influence of the Training

Overall, there was no significant difference in performances between the dogs trained with asymptomatic samples and those trained with symptomatic samples on Delta samples (sensitivities: 72.5% [57.7–82.8], *p* ≤ 0.0001, Chi-square, vs. 70.0% [48–86.9], *p* ≤ 0.0001 (Chi-square), *p* = 0.8188 (Friedman test with Bonferroni correction). The respective specificities were 54.3% [47.4–61.2] and 46.0% [38.0–54.0], *p* = 0.1265 (Friedman test with Bonferroni correction).

On Omicron samples, dogs trained on asymptomatic samples showed slightly better performance (sensitivities: 35.0% [13.5–62.2], *p* = 0.0422, Chi-square, vs. 20.0% [0.0–40.2], *p* = 0.3710, Chi-square). However, no significant differences were detected (*p* = 0.3310, Friedman test with Bonferroni correction). Given the small group sizes, these results may have been influenced by interindividual variability. Specificity was 55.0% [45.3–64.8] vs. 54.7% [43.4–65.9] with no significant difference (*p* = 0.9651, Friedman test with Bonferroni correction).

### 3.4. Influence of the Vaccination Status

For Delta samples, dog performance according to training type (S vs. AS) was not significantly different after adjustment for patients’ vaccination status (*p* = 0.8121).

Among vaccinated patients, the sensitivity of dogs trained on asymptomatic patients was 81.3% [54.4–96.0] compared to 81.8% [48.2–97.7] for dogs trained on symptomatic patients (RR = 0.99 [0.69–1.43]).

Among non-vaccinated patients, the sensitivity of dogs trained on asymptomatic patients was 66.7% [44.7–84.4] compared to 70.6% [44.0–89.7] for dogs trained on symptomatic patients (RR = 0.94 [0.62–1.43]).

Similarly, the sensitivity of dogs to detect vaccinated patients was not significantly different from their sensitivity to detect non-vaccinated patients, after adjustment for training type (*p* = 0.2339).

### 3.5. Influence of the Symptomatic/Asymptomatic Status

For Delta samples, dog performance according to dog training type (trained on symptomatic vs. asymptomatic samples) was not significantly different after adjustment for patients’ symptomatic status (*p* = 0.8199).

Among asymptomatic patient samples, the sensitivity for dogs trained on asymptomatic patients was 80.0% [56.3–94.3], compared with 73.3% [44.9–92.2] for dogs trained on symptomatic patients (RR = 1.09 [0.75–1.59]).

Among symptomatic patient samples, the sensitivity for dogs trained on asymptomatic patients was 65.0% [40.0–84.6], compared with 66.75 [33.4–88.2] for dogs trained on symptomatic patients (RR = 0.98 [0.60–1.58]).

Similarly, the sensitivity of dogs to detect asymptomatic patients was not significantly different from their sensitivity to detect symptomatic patients after adjustment for dog training type (*p* = 0.2968).

## 4. Discussion

This study explored whether dogs trained on samples from symptomatic COVID-19 patients would detect asymptomatic cases, and vice versa, a question that had not previously been examined. Consistent with a proof-of-concept approach, we did not advance a directional hypothesis regarding symptom status, but evaluated whether generalization across symptomatic and asymptomatic infections was feasible. In contrast, based on emerging evidence that SARS-CoV-2 variants may alter volatile organic compound profiles, we expected that detection performance might decline with newer viral variants such as Omicron. Our results supported the proof-of-concept approach: dogs trained on symptomatic patients successfully detected asymptomatic cases, and vice versa, for the Delta variant, showing that asymptomatic patients can be detected by dogs. However, as predicted, detection performance was reduced for Omicron samples from vaccinated individuals, suggesting that variant-specific and host-related factors constrain the generalizability of canine detection.

In our study, the yes/no procedure proved less suited for COVID-19 detection than the line-up design. Discrimination in a single-sample presentation scenario proved challenging for several reasons: the lack of paired samples from the same individuals (both sick and healthy), the potential reinforcement of visual targeting rather than olfaction, and the possibility that the dogs learned stimulus–response associations for specific samples (sit vs. stand-stare), a form of exemplar memorization that can occur when odor cues are weak or heterogeneous rather than forming a generalizable odor category. Although the dogs had previously succeeded in training with some human scents during the training phase of the yes/no procedure, identifying new COVID-19 samples may not have been sufficiently salient or specific for reliable detection. Importantly, reaching an accuracy criterion during training should not be interpreted as evidence of stable or transferable performance beyond the experimental context, as detection accuracy may fluctuate over time and across conditions. Similar concerns regarding the interpretation of canine detection performance and readiness for deployment have been raised by Edwards et al. [23], who proposed a go/no-go decision framework emphasizing the distinction between laboratory performance and operational validity.

More generally, the detection of human diseases by dogs relies entirely on operant conditioning, as disease-related odors have no intrinsic biological or ethological reward value for the animal. As a consequence, performance stability depends on the strength and maintenance of learned associations rather than on naturally salient stimuli. This limitation applies broadly to canine detection of human diseases and may partially explain the variability in performance observed across studies, as well as the difficulty in maintaining high detection accuracy over time or across contexts.

An additional limitation of the present study is that although responses were categorized as true positives, true negatives, false positives, and false negatives for the calculation of sensitivity and specificity, we did not further analyze error patterns (e.g., whether individual dogs tended to produce more false positives versus false negatives). Such analyses would require systematic video-based behavioral coding, which was beyond the scope of the present work. Future studies incorporating detailed error profiling may help disentangle effects related to training paradigms from those related to odor cues and provide further insight into individual response strategies. Also note that because each dog contributes many trials and repeatedly encounters some of the same Normal samples, the design is highly clustered and at risk of pseudoreplication. Chi-square tests may inflate the apparent precision of the estimates.

By contrast, the line-up procedure provided a more effective framework for this task. Presenting dogs with multiple samples simultaneously reduced the risk of location memorization and visual cueing, and allowed for relative discrimination of target vs. control odors. Under this protocol, dogs performed above chance when discriminating symptomatic and asymptomatic Delta samples, highlighting the importance of protocol selection in canine scent-detection studies. This shift in approach aimed at addressing the limitations encountered during the previous protocol, focusing on aspects of training and memory.

The eight dogs underwent two days of training using the same set of samples, this time presented in a lineup format. Remarkably, all dogs quickly achieved a success rate above 90%. The baseline assessment thus revealed high success rates. In Test 1 (new Delta samples), six out of eight dogs succeeded while two did not. There was no discernible difference in performance between samples from symptomatic versus asymptomatic patients. In Test 2 (Omicron samples, all asymptomatic and vaccinated), only three dogs managed to locate 5 out of 40 Omicron COVID-19 samples, resulting in a significant drop in overall performance.

In a lineup setup, dogs could compare the samples directly, without relying on a memorized odor, and promptly identify the most salient one. Trained dogs demonstrated the potential to achieve high success rates with minimal training, consistent with other studies. This was true regardless of whether a patient had COVID-19 symptoms.

More than twenty studies have been published on the ability of scent-detection dogs to detect COVID-19 [10]. In this systematic review, high risks of bias and problems with applicability and/or quality were noted. Only four studies were assessed as low risk of bias, and six as high quality. The four unbiased QUADAS-2 studies reported sensitivity ranges of 81–97% and specificity ranges of 91–100%. The six high-quality studies, according to the overall assessment system, reported sensitivity ranges of 82–97% and specificity ranges of 83–100%. Performance in our study was lower than in the literature. However, most other studies focused exclusively on highly symptomatic patients with pulmonary symptoms and on variants prior to Omicron.

While successful identification of COVID-19 samples in a line-up may reflect category learning based on repeated exposure to infected samples, it does not in itself demonstrate specificity to SARS-CoV-2-related volatile organic compounds. Importantly, individual Normal samples may be highly salient in terms of odor intensity or VOC composition, and salience alone does not necessarily correspond to disease status. These considerations motivate the need for disease control groups, discussed below, to distinguish pathogen-specific odor signatures from more general illness-related cues.

A key limitation of the present study is the absence of a disease control group consisting of individuals ill with non-COVID conditions. Without such controls, it is not possible to conclude with certainty that dogs were detecting VOCs specific to SARS-CoV-2 infection rather than more general markers of altered health. However, the inclusion of asymptomatic SARS-CoV-2-positive individuals partially addresses this concern, as these participants were infected but not clinically unwell. The ability of dogs to detect asymptomatic Delta samples suggests that detection was not solely driven by overt signs of illness, although specificity to COVID-19 cannot be definitely established. Future studies incorporating disease control groups will be essential to disentangle COVID-19-specific odor signatures from broader illness-related olfactory cues.

Our study was conducted at the end of the Delta wave and at the emergence of Omicron, when infection levels were extremely high. The kinetics of volatile compound emissions during SARS-CoV-2 infection remain completely unknown, as does the persistence of these compounds in the living spaces where samples were collected (hospital and home). Questions arise about the persistence of volatile compounds in the healthy population we selected (see [16]). Healthy individuals may not have developed symptoms of COVID-19 within seven days, but given the high incidence of the virus at that time, it is possible that some were asymptomatic COVID-19 carriers, which may have disrupted the odors detected by the dogs (InfoCovidFrance from 11 May 2020 to 30 June 2023 [24]). Longitudinal studies are needed to better estimate potential false negatives during an epidemic phase.

While the present study was conducted in the context of the COVID-19 pandemic, its rationale extends beyond SARS-CoV-2 itself. At a basic research level, the question addressed here, whether dogs trained on one clinical presentation of infection can generalize to another (e.g., symptomatic versus asymptomatic infection), remains central to understanding the limits of odor-based disease detection. Similar questions could be investigated for other viral infections, such as influenza.

At an applied research level, the present results should be considered as methodological building blocks for future needs. Emerging and re-emerging infectious diseases are expected to increase in frequency as a consequence of global environmental change. Studies such as the present one contribute to defining the conditions under which animal-based detection may or may not generalize across pathogens, variants, or clinical presentations.

From an epidemiological standpoint, new COVID-19 variants with different symptom profiles, severity, and viral loads may require regular retraining of detection dogs with the currently circulating variant. When exposed to samples of a new variant (Omicron), performance was not maintained under the present conditions, with dogs showing reduced detection accuracy. This suggests that the olfactory signature may have differed from that of Delta or may have been less salient under the present conditions. Omicron infections were generally milder, especially in the lungs, than Delta infections, which may have influenced volatile compound release [25,26].

Only one other study tested dogs with both the Delta and Omicron variants. Mutesa et al. [27] reported a clear decrease in performance with Omicron compared to Delta. They also observed higher Ct values for the Omicron variant, suggesting lower viral loads, which may partly explain the poorer detection rates.

One potential limitation of the Omicron test phase is that Omicron-positive samples were presented in line-ups with Normal samples that had been encountered previously during the Delta test phase, rather than with entirely novel Normal samples. However, because line-ups were randomized across dogs and trials, not all Normal samples had necessarily been encountered previously: some were new, others repeated. This introduces a possible novelty asymmetry between target and distractor odors. Ideally, a fully independent set of Normal samples would have been used to eliminate any potential novelty-related confounds. Importantly, however, such an asymmetry would be expected to facilitate, rather than impair, detection of the novel target odor. The observed reduction in performance for Omicron samples, therefore, cannot be readily explained by novelty effects alone and is more consistent with a genuine alteration in odor profiles associated with the Omicron variant and/or host-related factors. Vaccination also appears to alter viral load dynamics, accelerating clearance and potentially modifying volatile compound production. Our findings also align closely with [15], as dogs trained on Delta did not maintain performance when tested with Omicron samples from vaccinated individuals, supporting the view that variant- and host-related factors can alter odor signatures and limit generalizability. In September 2021, half of the French population was fully vaccinated. Several studies reported reduced viral load and faster clearance in vaccinated individuals [10,23,24,25,26,27,28,29,30,31,32,33]. In our study, more than two-thirds of participants were vaccinated, which may have reduced the strength of volatile odor cues.

Taken together, these results should be interpreted with caution. The Omicron test phase included only five SARS-CoV-2-positive individuals, all of whom were asymptomatic and vaccinated, and detection performance did not differ significantly from chance. Although the pattern of results is consistent with a potential decline in detection performance for Omicron in vaccinated individuals, the small sample size and remaining design constraints (including novelty asymmetry between target and distractor samples) preclude strong conclusions. These findings should therefore be considered preliminary and highlight the need for further studies using larger sample sizes, disease control groups, and fully independent distractor sets.

Note that during the pandemic, a drastic reduction in other viral (influenza, respiratory syncytial virus, etc.) or bacterial (Streptococcus pneumoniae, etc.) infectious was observed, due to the measures taken to mitigate the transmission of COVID-19 (face masks, hand hygiene, social distancing, screening and isolation of sick individuals, etc.), but also due to virus-specific transmission factors, such as viral interference (direct or indirect antagonistic interaction between respiratory viruses that affects the ability of a virus to infect and cause disease in the host). It was therefore not feasible to establish a control group with odors from subjects infected with other types of germs in the respiratory tract.

This study has several strengths. It was a randomized, double-blind, prospective design using a robust protocol: handlers were blinded, and the originally fully non-working dogs worked in a room without humans present. Eight dogs were trained with positive reinforcement, including two groups specifically trained with asymptomatic or symptomatic samples. Sample collection was standardized and performed by the same individual, and both home and hospital samples were represented across groups (symptomatic, asymptomatic, and controls), minimizing location bias. Importantly, all test samples were novel compared with training samples, and no scent lines were repeated, avoiding the artificial inflation of sensitivity sometimes seen in studies where dogs sniff the same samples multiple times. COVID-19 patients without respiratory symptoms were also included. Detailed, individual-level data were collected for each dog, further strengthening the reliability of the study.

Nevertheless, some limitations should be acknowledged. The dogs were relatively inexperienced (“green”), unlike other studies that relied on highly trained explosives-detection dogs. The protocol was also modified mid-study, moving from a yes/no to a line-up procedure, which may have introduced variability in training history, but also a pre-history in detecting COVID-19 scent. The Omicron test phase must be interpreted with particular caution. Only five SARS-CoV-2-positive individuals were included, all of whom were asymptomatic and vaccinated, which severely limits statistical power and precludes strong inference about generalization across variants. Importantly, variant, vaccination, status, and symptom profile were fully confounded in this phase. As a result, it is not possible to determine whether the observed reduction in detection performance reflects variant-specific changes in VOC profiles, vaccination-related effects on viral load or host metabolism, characteristics of asymptomatic infection, or random variation associated with a very small sample size. Accordingly, the Omicron findings should be considered exploratory and hypothesis-generating rather than evidence of true variant-specific odor changes.

## 5. Conclusions

This study provides the first controlled evidence that dogs trained on symptomatic COVID-19 patients can detect asymptomatic cases, and vice versa, at least for the Delta variant and in a line-up protocol. However, performance was markedly reduced for Omicron samples from vaccinated individuals, highlighting the dependence of canine detection on viral variant and host immune status.

These findings underline both the potential and the limitations of medical detection dogs in infectious disease surveillance. Dogs can generalize across symptom status under controlled conditions, but their reliability is compromised by evolving viral biology and population-level vaccination. Future research should focus on (1) standardizing protocols, (2) clarifying the biological origin of VOC signatures, and (3) assessing whether frequent retraining with circulating variants (for influenza, for instance, not SARS-CoV-2, given its decrease) can sustain diagnostic accuracy. Until such evidence is available, the use of canine detection for large-scale public health screening should be considered exploratory and not a substitute for conventional testing for current or future infectious diseases.

## Figures and Tables

**Table 1:** Characteristics of dog participants.

Dog	Breed	Age in Years	Sex
Blaike	Mix breed	7	Male
Ghost	Australian shepherd mix	2	Male
Lina	Mix breed	7	Female
Orco	Labrador	2	Male
Icare	Jack Russell Terrier	9	Male
Peps	Shepherd mix	2	Male
Perle	Labradoodle	2	Female
Ramses	Labrador	1.5	Male

**Table 2:** Repartition of the type of stimuli according to the eight dogs.

	ANIMALIN				ACADIA			
	Blaike	Lina	Icare	Ramses	Ghost	Orco	Peps	Perle
N = 20	20	20	20	20	20	20	20	20
AS = 10	10	10			10	10		
S = 10			10	10			10	10

**Table 3:** Correct response rate broken down by dog (Delta).

Dogs	Type	Sample Size	Correct Answers (%)	Sen *	*p*
BLAIKE	Asympto	60	28 (46.7%)	70%	0.0093
GHOST	Asympto	60	33 (55.0%)	50%	
LINA	Asympto	59	37 (62.7%)	90%	
ORCO	Asympto	60	39 (65.0%)	80%	
PEPS	Sympto	60	29 (48.3%)	70%	
PERLE	Sympto	60	22 (36.7%)	60%	
RAMSES	Sympto	60	39 (65.0%)	80%	
All (*n* = 7)		419	227 (54.2%)	N/A	N/A

* Sensitivity calculated on 10 samples (10 patients).

**Table 4:** Correct response rate broken down by dog (Omicron).

Dog	Type	Sample Size	Correct Answers (%)	Sen *	*p*
BLAIKE	Asympto	30	13 (43.3%)	20.0%	0.0014
GHOST	Asympto	30	8 (26.7%)	40.0%	
LINA	Asympto	30	16 (53.3%)	0%	
ORCO	Asympto	30	25 (83.3%)	80.0%	
PEPS	Sympto	30	14 (46.7%)	0%	
PERLE	Sympto	30	13 (43.3%)	20%	
RAMSES	Sympto	30	17 (56.7%)	40%	
All (*n* = 7)		210	106 (50.5%)	N/A	N/A

* Sensitivity calculated on 5 samples (5 patients).

## Data Availability

Datasets are accessible in the Open Science Framework repository at the following link: https://osf.io/h2cf3/overview?view_only=b298a13e72e24195a92b34363e5c0b92 (accessed on 28 October 2025).

## Supplementary Materials

The following supporting information can be downloaded at https://www.mdpi.com/article/10.3390/ani16030480/s1.

## References

[1] Reeve C., Wilson C., Hanna D., Gadbois S. (2021). Dog Owners’ Survey reveals Medical Alert Dogs can alert to multiple conditions and multiple people. PLoS ONE.

[2] Catala A., Grandgeorge M., Schaff J.L., Cousillas H., Hausberger M., Cattet J. (2019). Dogs demonstrate the existence of an epileptic seizure odour in humans. Sci. Rep..

[3] Hardin D.S., Anderson W., Cattet J. (2015). Dogs can be successfully trained to alert to hypoglycemia samples from patients with type 1 diabetes. Diabetes Ther..

[4] Bauër P., Leemans M., Audureau E., Gilbert C., Armal C., Fromantin I. (2022). Remote medical scent detection of cancer and infectious diseases with dogs and rats: A systematic review. Integr. Cancer Ther..

[5] Angle T.C., Passler T., Waggoner P.L., Fischer T.D., Rogers B., Galik P.K. (2015). Real-time detection of a virus using detection dogs. Front. Vet. Sci..

[6] Aksenov A.A., Sandrock C.E., Zhao W., Sankaran S., Schivo M., Harper R., Cardona C.J., Xing Z., Davis C.E. (2014). Cellular scent of influenza virus infection. Chembiochem.

[7] Otto C.M., Sell T.K., Veenema T.G., Hosangadi D., Vahey R.A., Connell N.D., Privor-Dumm L. (2023). The promise of disease detection dogs in pandemic response: Lessons learned from COVID-19. Disaster Med. Public Health Prep..

[8] Gadbois S., Reeve C. (2014). Canine olfaction: Scent, sign, and situation. Domestic Dog Cognition and Behavior: The Scientific Study of Canis familiaris.

[9] Halfon P., Penaranda G., Khiri H., Garcia V., Drouet H., Philibert P., Psomas C., Delord M., Retornaz F., Allardet-Servent J. (2021). An optimized stepwise algorithm combining rapid antigen and RT-qPCR for screening of COVID-19 patients. PLoS ONE.

[10] Meller S., Caraguel C., Twele F., Charalambous M., Schoneberg C., Chaber A.L., Desquilbet L., Grandjean D., Mardones F.O., Kreienbrock L. (2023). Canine olfactory detection of SARS-CoV-2-infected humans-a systematic review. Annu. Epidemiol..

[11] Guest C., Pinder M., Doggett M., Squires C., Affara M., Kandeh B., Dewhirst S., Morant S.V., D’ALessandro U., Lindsay S.W. (2019). Trained dogs identify people with malaria parasites by their odour. Lancet Infect. Dis..

[12] Guest C., Dewhirst S.Y., Allen D.J., Aziz S., Baerenbold O., Bradley J. (2022). Using trained dogs and organic semi-conducting sensors to identify asymptomatic and mild SARS-CoV-2 infections. J. Travel Med..

[13] Grandjean D., Elie C., Gallet C., Julien C., Roger V., Desquilbet L., Alvergnat G., Delarue S., Gabassi A., Treluyer J.M. (2022). Diagnostic accuracy of non-invasive detection of SARS-CoV-2 infection by canine olfaction. PLoS ONE.

[14] Bax C., Robbiani S., Zannin E., Capelli L., Ratti C., Bonetti S., Novelli L., Raimondi F., Di Marco F., Dellacà R.L. (2022). An experimental apparatus for e-nose breath analysis in respiratory failure patients. Diagnostics.

[15] Ungar P.J., Pellin M.A., Malone L.A. (2023). A One Health perspective: COVID-sniffing dogs can be effective and efficient as public health guardians. J. Am. Vet. Med. Assoc..

[16] Ozgur-Buyukatalay E., Demirbas Y.S., Bozdayi G., Kismali G., Ilhan M.N. (2023). Is diagnostic performance of SARS-CoV-2 detection dogs reduced-due to virus variation-over the time?. Appl. Anim. Behav. Sci..

[17] Grizzi F., Bax C., Farina F.M., Tidu L., Hegazi M.A., Chiriva-Internati M., Capelli L., Robbiani S., Dellacà R., Taverna G. (2024). Recapitulating COVID-19 detection methods: RT-PCR, sniffer dogs and electronic nose. Diagn. Microbiol. Infect. Dis..

[18] Van Doremalen N., Bushmaker T., Morris D.H., Holbrook M.G., Gamble A., Williamson B.N., Tamin A., Harcourt J.L., Thornburg N.J., Munster V.J. (2020). Aerosol and surface stability of SARS-CoV-2 as compared with SARS-CoV-1. N. Engl. J. Med..

[19] Duranton C., Bedossa T., Gaunet F. (2019). Pet dogs exhibit social preference for people who synchronize with them: What does it tell us about the evolution of behavioral synchronization?. Anim. Cogn..

[20] Gadbois S., Reeve C. (2016). The semiotic canine: Scent processing dogs as research assistants in biomedical and environmental research. Dog Behav..

[21] Skinner B.F. (1938). The Behavior of Organisms: An Experimental Analysis.

[22] Thorndike E. (1898). Some experiments on animal intelligence. Science.

[23] Edwards T.L., Browne C.M., Schoon A., Cox C., Poling A. (2017). Animal olfactory detection of human diseases: Guidelines and systematic review. J. Vet. Behav..

[24] InfoCovidFrance on Santé Public France. https://www.santepubliquefrance.fr/dossiers/coronavirus-covid-19/coronavirus-chiffres-cles-et-evolution-de-la-covid-19-en-france-et-dans-le-monde.

[25] Relan P., Motaze N.V., Kothari K., Askie L., de Waroux O.L.P., Van Kerkhove M.D., Diaz J., Vijayaraghavan B.K.T. (2023). Severity and outcomes of Omicron variant of SARS-CoV-2 compared to Delta variant and severity of Omicron sublineages: A systematic review and metanalysis. BMJ Glob. Health.

[26] Mendiola-Pastrana I.R., López-Ortiz E., Río de la Loza-Zamora J.G., González J., Gómez-García A., López-Ortiz G. (2022). SARS-CoV-2 variants and clinical outcomes: A systematic review. Life.

[27] Mutesa L., Misbah G., Remera E., Ebbers H., Schalke E., Tuyisenge P., Sindayiheba R., Igiraneza C., Uwimana J., Nsanzimana S. (2022). Use of trained scent dogs for detection of COVID-19 and evidence of cost-saving. Front. Med..

[28] Janes H., Fisher L.H., Kee J.J., Parameswaran L., Goepfert P.A., Falsey A.R., Ludwig J., AMagaret C., Gilbert P.B., Huang Y. (2024). Association between SARS-CoV-2 viral load and COVID-19 vaccination in 4 phase 3 trials. J. Infect. Disseases.

[29] Coppeta L., Balbi O., Grattagliano Z., Mina G.G., Pietroiusti A., Magrini A., Bolcato M., Aurilio M.T. (2021). First dose of the BNT162b2 mRNA COVID-19 vaccine reduces symptom duration and viral clearance in healthcare workers. Vaccines.

[30] Andeweg S.P., van de Kassteele J., Wang X., van Maarseveen N., Vlaemynck B., Bos S., Vennema H., Presser L., Cai J.J., Eggink D. (2025). Estimating the effect of COVID-19 vaccination and prior infection on cycle threshold values as a proxy of SARS-CoV-2 viral load. Int. J. Infect. Dis..

[31] Lunt R., Quinot C., Kirsebom F., Andrews N., Skarnes C., Letley L., Haskins D., Angel C., Firminger S., Ratcliffe K. (2024). The impact of vaccination and SARS-CoV-2 variants on the virological response to SARS-CoV-2 infections during the Alpha, Delta, and Omicron waves in England. J. Infect..

[32] Woodbridge Y., Amit S., Huppert A., Kopelman N.M. (2022). Viral load dynamics of SARS-CoV-2 Delta and Omicron variants following multiple vaccine doses and previous infection. Nat. Commun..

[33] Qassim S.H., Hasan M.R., Tang P., Chemaitelly H., Ayoub H.H., Yassine H.M., Al-Khatib H.A., Smatti M.K., Abdul-Rahim H.F., Abu-Raddad L.J. (2022). Effects of SARS-CoV-2 Alpha, Beta, and Delta variants, age, vaccination, and prior infection on infectiousness of SARS-CoV-2 infections. Front. Immunol..

