# Supplementary material for: Dogs’ Detection of Symptomatic and Asymptomatic SARS-CoV-2 by Non-Working Dogs: Feasibility and Limits Under Controlled Laboratory Conditions
Source: Animals (Basel). 2026 Feb 4;16(3):480. doi: 10.3390/ani16030480 (PMC12896432; doi:10.3390/ani16030480)
Supplement: Supplementary file 1 [file animals-16-00480-s001.zip › animals-3985586-supplementary.pdf]

animals

MDPI

Article

Dogs’ Detection of Symptomatic and Asymptomatic SARS-CoV-2 by Non-Working Dogs: Feasibility and Limits Under Controlled Laboratory Conditions

Jennifer Cattet [1,†], Frédérique Retornaz [2], Florine Munier [3,‡], Catherine Collignon [4,‡] and Florence Gaunet [5,*,†]

[1] Center for the Study of Medical Alert Canines (CMAC), and Medical Mutts Service Dogs, 6120 Allisonville Rd, Indianapolis, IN 46220, USA; jen@medical-mutts.com
[2] Unité de Soins et de Recherche en Médecine Interne et Maladies Infectieuses, Hôpital Européen, 6 Rue Désirée Clary, 13003 Marseille, France; f.retornaz@hopital-europeen.fr
[3] Chiens D'assistance Pour Diabétiques Centre Domazane (ACADIA), 295 Chemin des Buis, 26250 Livron sur Drôme, France; florine.munier@acadia-asso.org
[4] ANIMALIN, 173 Rue des jardins, 32450 Aurimont, France; info@centreanimalin.fr
[5] Centre de Recherche en Psychologie et Neurosciences (UMR 7077), Aix-Marseille University and CNRS, 3, Place Victor Hugo, 13003 Marseille, France
* Correspondence: florence.gaunet@univ-amu.fr
† These authors contributed equally to this work.
‡ These authors also contributed equally to this work.

SAMPLE NUMBER	Age	Gender	Collection location	Group assigned	Cough	Anosmia	Diarrhea	Fever	Vaccination	Vaccination _ number of doses	Type of Vaccine	Body mass Index	Active smoking	Diabetes	High Blood Pressure	Dyslipidemia	Ischemic cardiopathy	Cancer	Severe renal failure	Cirrhosis	Pregnancy
	years	Women/ Men	Hospital/Home	Asymptomatic /Symptomatic /	YES/NO	YES/NO	YES/NO	YES/NO	YES/NO	0/1/2/3		kg/M2	YES/NO	YES/NO	YES/NO	YES/NO	YES/NO	YES/NO	YES/NO	YES/NO	YES/NO

 https://doi.org/10.3390/ani16030480

				Healthy Control																	
LN1	24	W	Hospital	Healthy Control	NO	NO	NO	NO	YES	2	PFIZER	24.1	NO	NO	NO	NO	NO	NO	NO	NO	NO
LN2	51	W	Hospital	Healthy Control	NO	NO	NO	NO	YES	2	PFIZER	20.7	NO	NO	NO	NO	NO	NO	NO	NO	NO
LN3	29	M	Hospital	Healthy Control	NO	NO	NO	NO	YES	2	PFIZER	20.5	NO	YES	NO	NO	NO	NO	NO	NO	NO
LN4	59	W	Hospital	Healthy Control	NO	NO	NO	NO	YES	2	PFIZER	21.3	NO	NO	YES	NO	NO	NO	NO	NO	NO
LN5	58	W	Home	Healthy Control	NO	NO	NO	NO	YES	2	PFIZER	20.1	NO	NO	NO	NO	NO	NO	NO	NO	NO
LN6	57	W	Hospital	Healthy Control	NO	NO	NO	NO	YES	2	PFIZER	21.8	YES	NO	NO	NO	NO	NO	NO	NO	NO
LN7	21	W	Hospital	Healthy Control	NO	NO	NO	NO	YES	1	PFIZER	23.6	NO	NO	NO	NO	NO	NO	NO	NO	NO
LN8	40	W	Hospital	Healthy Control	NO	NO	NO	NO	YES	2	PFIZER	22.3	YES	NO	NO	NO	NO	NO	NO	NO	NO
LN9	24	W	Hospital	Healthy Control	NO	NO	NO	NO	YES	2	ASTRA-ZENECA / PFIZER	27.0	YES	NO	NO	NO	NO	NO	NO	NO	NO
LN10	44	M	Hospital	Healthy Control	NO	NO	NO	NO	YES	2	PFIZER	23.6	NO	NO	NO	NO	NO	NO	NO	NO	NO
LN11	27	W	Hospital	Healthy Control	NO	NO	NO	NO	YES	2	ASTRA-ZENECA / PFIZER	26.3	NO	NO	NO	NO	NO	NO	NO	NO	NO
LN12	42	W	Hospital	Healthy Control	NO	NO	NO	NO	YES	2	PFIZER	21.4	NO	NO	NO	NO	NO	NO	NO	NO	NO

https://doi.org/10.3390/ani16030480

LN13	45	M	Hospital	Healthy Control	NO	NO	NO	NO	YES	2	PFIZER	30.7	NO	NO	NO	NO	NO	NO	NO	NO	NO
LN14	21	W	Hospital	Healthy Control	NO	NO	NO	NO	NO	0	NA	19.4	NO	NO	NO	NO	NO	NO	NO	NO	NO
LN15	36	M	Hospital	Healthy Control	NO	NO	NO	NO	YES	1	PFIZER	28.1	NO	NO	NO	NO	NO	NO	NO	NO	NO
LN16	51	W	Hospital	Healthy Control	NO	NO	NO	NO	YES	2	PFIZER	28.0	NO	NO	NO	NO	NO	NO	NO	NO	NO
LN17	57	M	Home	Healthy Control	NO	NO	NO	NO	YES	2	PFIZER	32.9	NO	NO	NO	NO	NO	NO	NO	NO	NO
LN18	38	W	Hospital	Healthy Control	NO	NO	NO	NO	YES	2	PFIZER	21.0	NO	NO	NO	NO	NO	NO	NO	NO	NO
LN19	24	W	Hospital	Healthy Control	NO	NO	NO	NO	YES	1	PFIZER	21.5	YES	NO	NO	NO	NO	NO	NO	NO	NO
LN20	56	W	Hospital	Healthy Control	NO	NO	NO	NO	YES	2	PFIZER	21.3	NO	NO	NO	NO	NO	NO	NO	NO	NO
LS1	50	M	Hospital	Symptomatic	YES	YES	NO	YES	NO	0	NA	21.8	NO	NO	NO	NO	NO	NO	NO	NO	NO
LS2	27	M	Home	Symptomatic	YES	NO	NO	YES	YES	2	PFIZER	22.7	NO	NO	NO	NO	NO	NO	NO	NO	NO
LS3	68	W	Hospital	Symptomatic	YES	YES	NO	YES	NO	0	NA	33.2	NO	NO	YES	NO	NO	NO	NO	NO	NO
LS4	57	W	Hospital	Symptomatic	YES	NO	NO	YES	NO	0	NA	31.6	NO	YES	YES	NO	NO	NO	NO	NO	NO
LS5	61	M	Hospital	Symptomatic	YES	NO	NO	YES	NO	0	NA	25.8	NO	YES	NO	NO	NO	NO	NO	NO	NO
LS6	19	W	Home	Symptomatic	YES	NO	NO	YES	NO	0	NA	27.7	NO	NO	NO	NO	NO	NO	NO	NO	NO
LS7	20	W	Home	Symptomatic	NO	YES	NO	NO	NO	0	NA	19.1	YES	NO	NO	NO	NO	NO	NO	NO	NO
LS8	29	W	Hospital	Symptomatic	YES	NO	NO	YES	NO	0	NA	17.2	YES	NO	NO	NO	NO	NO	NO	NO	NO
LS9	38	M	Hospital	Symptomatic	YES	NO	NO	YES	NO	0	NA	24.4	NO	NO	NO	NO	NO	NO	NO	NO	NO
LS10	72	W	Hospital	Symptomatic	YES	NO	NO	YES	NO	0	NA	27.3	NO	NO	YES	NO	NO	NO	NO	NO	NO
LAS1	31	M	Home	Asymptomatic	NO	NO	NO	NO	YES	2	PFIZER	24.2	NO	NO	NO	NO	NO	NO	NO	NO	NO

https://doi.org/10.3390/ani16030480

LAS2	60	W	Hospital	Asymptomatic	NO	NO	NO	NO	YES	2	PFIZER	33.2	YES	NO	YES	YES	YES	NO	NO	NO	NO
LAS3	21	W	Hospital	Asymptomatic	NO	NO	NO	NO	NO	0	NA	33.1	NO	NO	NO	NO	NO	NO	NO	NO	NO
LAS4	78	M	Hospital	Asymptomatic	NO	NO	NO	NO	YES	1	PFIZER	23.4	NO	YES	YES	YES	YES	NO	NO	NO	NO
LASS	35	W	Home	Asymptomatic	NO	NO	NO	NO	YES	1	PFIZER	21.6	NO	NO	NO	NO	NO	NO	NO	NO	YES
LAS6	78	M	Hospital	Asymptomatic	NO	NO	NO	NO	YES	2	PFIZER	22.2	YES	NO	YES	NO	NO	NO	NO	NO	NO
LAS7	79	M	Hospital	Asymptomatic	NO	NO	NO	NO	YES	2	SINOPHARMA	34.0	NO	NO	YES	NO	NO	NO	NO	NO	NO
LAS8	62	M	Home	Asymptomatic	NO	NO	NO	NO	YES	1	JANSSEN	19.0	YES	NO	NO	NO	NO	NO	NO	NO	NO
LAS9	91	W	Hospital	Asymptomatic	NO	NO	NO	NO	YES	2	PFIZER	21.1	NO	NO	YES	NO	NO	NO	NO	NO	NO
LAS10	45	M	Home	Asymptomatic	NO	NO	NO	NO	YES	2	PFIZER	20.9	NO	NO	NO	NO	NO	NO	NO	NO	NO

Part I. Human participants characteristics of scent stimuli for the Yes/No protocol: learning phase of Delta samples**.**

Odor samples of human participants LN1 to LN20 (controls in green), LS1 to LS10 (symptomatics in orange) and LAS1 to LAS10 (asymptomatics in yellow) were used in both schools.

SAMPLE NUMBER	Age	Gender	Collection location	Group assigned	Cough	Anosmia	Diarrhea	Fever	Vaccination	Vaccination_	Type of Vac	Bodymass	Active smoking	Diabetes	High Blood	Dyslipidemia	Ischemic	Cancer	Severe renal	Cirrhosis	Pregnancy

										number oW doses	cine	Index			Pressure		cardiopathy		failure		
		Women/Men	Hospital/Home	Asymptomatic /Symptomatic / Healthy Control	YES/NO	YES/NO	YES/NO	YES/NO	YES/NO	0/1/2/3		kg/M2	YES/NO	YES/NO	YES/NO	YES/NO	YES/NO	YES/NO	YES/NO	YES/NO	YES/NO
LN21	45	M	Hospital	Healthy Control	NO	NO	NO	NO	YES	2	PFIZER	22.2	YES	NO	NO	NO	NO	NO	NO	NO	NO
LN22	24	W	Hospital	Healthy Control	NO	NO	NO	NO	YES	2	PFIZER	22.5	NO	NO	NO	NO	NO	NO	NO	NO	NO
LN23	32	W	Hospital	Healthy Control	NO	NO	NO	NO	YES	2	PFIZER	25.0	NO	NO	NO	NO	NO	NO	NO	NO	NO
LN24	29	W	Hospital	Healthy Control	NO	NO	NO	NO	YES	2	PFIZER	21.1	NO	NO	NO	NO	NO	NO	NO	NO	NO
LN25	45	W	Hospital	Healthy Control	NO	NO	NO	NO	YES	2	PFIZER	25.0	NO	NO	NO	NO	NO	NO	NO	NO	NO

https://doi.org/10.3390/ani16030480

LN26	30	M	Hospital	Healthy Control	NO	NO	NO	NO	YES	2	PFIZER	26.3	YES	NO	NO	NO	NO	NO	NO	NO	NO
LN27	28	W	Hospital	Healthy Control	NO	NO	NO	NO	YES	2	PFIZER	22.5	YES	NO	NO	NO	NO	NO	NO	NO	NO
LN28	49	W	Hospital	Healthy Control	NO	NO	NO	NO	YES	1	PFIZER	23.1	NO	NO	NO	NO	NO	NO	NO	NO	NO
LN29	50	W	Hospital	Healthy Control	NO	NO	NO	NO	YES	2	PFIZER	36.2	NO	NO	YES	NO	NO	NO	NO	NO	NO
LN30	19	W	Hospital	Healthy Control	NO	NO	NO	NO	YES	2	PFIZER	23.6	NO	NO	NO	NO	NO	NO	NO	NO	NO
LS1*	50	M	Hospital	Symptomatic	YES	YES	NO	YES	NO	0	NA	21.8	NO	NO	NO	NO	NO	NO	NO	NO	NO
LS2 *	27	M	Home	Symptomatic	YES	NO	NO	YES	YES	2	PFIZER	22.7	NO	NO	NO	NO	NO	NO	NO	NO	NO
LS3*	68	W	Hospital	Symptomatic	YES	YES	NO	YES	NO	0	NA	33.2	NO	NO	YES	NO	NO	NO	NO	NO	NO
LS5 *	61	M	Hospital	Symptomatic	YES	NO	NO	YES	NO	0	NA	25.8	NO	YES	NO	NO	NO	NO	NO	NO	NO
LS7 *	20	W	Home	Symptomatic	NO	YES	NO	NO	NO	0	NA	19.1	YES	NO	NO	NO	NO	NO	NO	NO	NO

https://doi.org/10.3390/ani16030480

LAS1 *	31	M	Home	Asymptomatic	NO	NO	NO	NO	YES	2	PFIZER	24.2	NO	NO	NO	NO	NO	NO	NO	NO	NO
LAS2 *	60	W	Hospital	Asymptomatic	NO	NO	NO	NO	YES	2	PFIZER	33.2	YES	NO	YES	YES	YES	NO	NO	NO	NO
LAS3 *	21	W	Hospital	Asymptomatic	NO	NO	NO	NO	NO	0	NA	33.1	NO	NO	NO	NO	NO	NO	NO	NO	NO
LAS4 *	78	M	Hospital	Asymptomatic	NO	NO	NO	NO	YES	1	PFIZER	23.4	NO	YES	YES	YES	YES	NO	NO	NO	NO
LAS 5 *	35	W	Home	Asymptomatic	NO	NO	NO	NO	YES	1	PFIZER	21.6	NO	NO	NO	NO	NO	NO	NO	NO	YES

Part II. Human participants characteristics of scent stimuli for Line-up procedure: learning phase of Delta samples**.** Controls are in green, symptomatics in orange and asymptomatics in yellow.

SAMPLE NUMBER	Age	Gender	Collection location	Group assigned	Cough	Anosmia	Diarrhea	Fever	Vaccination	Vaccination_number of	Type of Vaccine	Body mass Index	Active smoking	Diabetes	High Blood Pressure	Dyslipidemia	Ischemic cardiopathy	Cancer	Severe renal failure	Cirrhosis	Pregnancy

										doses												
		Women/Men	Hospital/Home	Asymptomatic /Symptomatic / Healthy Control	YES/NO	YES/NO	YES/NO	YES/NO	YES/NO	0/1/2/3		kg/M2	YES/NO	YES/NO	YES/NO	YES/NO	YES/NO	YES/NO	YES/NO	YES/NO	YES/NO
TS10	57	M	Hospital	Symptomatic	YES	YES	YES	YES	NO	0	NA	30.4	NO	NO	NO	NO	NO	NO	NO	NO	NO
TS25	53	M	Hospital	Symptomatic	YES	YES	YES	YES	NO	0	NA	25.7	NO	NO	NO	NO	NO	NO	NO	NO	NO
TS27	61	W	Hospital	Symptomatic	YES	YES	YES	NO	NO	0	NA	23.3	NO	NO	NO	NO	NO	NO	NO	NO	NO
TS28	45	W	Hospital	Symptomatic	YES	YES	YES	YES	NO	0	NA	22.2	NO	NO	NO	NO	NO	NO	NO	NO	NO
TS29	48	M	Hospital	Symptomatic	YES	YES	NO	YES	NO	0	NA	25.1	NO	NO	NO	NO	NO	NO	NO	NO	NO
TAS6	71	W	Home	Asymptomatic	NO	NO	NO	NO	YES	2	PFIZER	32.0	NO	NO	YES	NO	NO	NO	NO	NO	NO
TAS11	21	M	Home	Asymptomatic	NO	NO	NO	NO	YES	2	PFIZER	18.4	NO	NO	NO	NO	NO	NO	NO	NO	NO
TAS12	22	M	Hospital	Asymptomatic	NO	NO	NO	NO	NO	0	NA	31.0	NO	NO	NO	NO	NO	NO	NO	NO	NO
TAS13	45	M	Home	Asymptomatic	NO	NO	NO	NO	YES	1	JANSSEN	24.9	NO	NO	NO	NO	NO	NO	NO	NO	NO
TAS15	47	W	Home	Asymptomatic	NO	NO	NO	NO	YES	2	PFIZER	19.6	NO	NO	NO	NO	NO	NO	NO	NO	NO

TN3*	28	W	Hospital	Healthy Control	NO	NO	NO	NO	NO	0	NA	21.0	YES	NO	NO	NO	NO	NO	NO	NO	NO
TN5*	54	W	Hospital	Healthy Control	NO	NO	NO	NO	NO	0	NA	20.8	YES	NO	NO	NO	NO	NO	NO	NO	NO
TN6*	59	W	Hospital	Healthy Control	NO	NO	NO	NO	YES	2	PFIZER	20.2	YES	NO	NO	NO	NO	NO	NO	NO	NO
TN9*	66	W	Hospital	Healthy Control	NO	NO	NO	NO	YES	2	PFIZER	28.8	YES	NO	YES	NO	NO	NO	NO	NO	NO
TN15*	52	W	Hospital	Healthy Control	NO	NO	NO	NO	YES	2	PFIZER	23.6	YES	NO	YES	NO	NO	NO	NO	NO	NO
TN16*	33	W	Hospital	Healthy Control	NO	NO	NO	NO	YES	2	PFIZER	23.4	YES	NO	NO	NO	NO	NO	NO	NO	NO
TN17*	35	W	Hospital	Healthy Control	NO	NO	NO	NO	YES	2	PFIZER	22.3	NO	NO	NO	NO	NO	NO	NO	NO	NO
TN18*	56	W	Hospital	Healthy Control	NO	NO	NO	NO	YES	2	PFIZER	29.7	YES	NO	NO	NO	NO	NO	NO	NO	NO
TN19*	22	M	Hospital	Healthy Control	NO	NO	NO	NO	YES	2	PFIZER	26.0	NO	NO	NO	NO	NO	NO	NO	NO	NO
TN20*	26	W	Hospital	Healthy Control	NO	NO	NO	NO	YES	1	PFIZER	23.4	YES	NO	YES	NO	NO	NO	NO	NO	NO
TN21*	32	W	Hospital	Healthy Control	NO	NO	NO	NO	YES	1	PFIZER	28.8	NO	NO	NO	NO	NO	NO	NO	NO	NO
TN22*	27	M	Hospital	Healthy Control	NO	NO	NO	NO	YES	2	PFIZER	19.8	YES	NO	NO	NO	NO	NO	NO	NO	NO
TN23*	55	W	Hospital	Healthy Control	NO	NO	NO	NO	YES	2	PFIZER	21.5	NO	NO	NO	NO	NO	NO	NO	NO	NO
TN24*	22	W	Hospital	Healthy Control	NO	NO	NO	NO	YES	1	PFIZER	28.3	NO	NO	NO	NO	NO	NO	NO	NO	NO

TN25*	52	W	Hospital	Healthy Control	NO	NO	NO	NO	YES	2	PFIZER	30.8	YES	NO	YES	YES	YES	NO	NO	NO	NO
TN26*	26	W	Hospital	Healthy Control	NO	NO	NO	NO	YES	1	ASTRA-ZENECA	26.2	NO	NO	NO	NO	NO	NO	NO	NO	NO
TN27*	24	W	Hospital	Healthy Control	NO	NO	NO	NO	YES	1	PFIZER	23.1	NO	NO	NO	NO	NO	NO	NO	NO	NO
TN28*	23	W	Hospital	Healthy Control	NO	NO	NO	NO	YES	2	PFIZER	24.7	YES	NO	NO	NO	NO	NO	NO	NO	NO
TN29*	20	W	Hospital	Healthy Control	NO	NO	NO	NO	YES	1	PFIZER	21.4	YES	NO	NO	NO	NO	NO	NO	NO	NO
TN30*	38	W	Hospital	Healthy Control	NO	NO	NO	NO	YES	2	PFIZER	19.9	NO	NO	NO	NO	NO	NO	NO	NO	NO
TN31*	35	M	Hospital	Healthy Control	NO	NO	NO	NO	YES	2	PFIZER	23.7	YES	NO	NO	NO	NO	NO	NO	NO	NO
TN33*	28	M	Hospital	Healthy Control	NO	NO	NO	NO	YES	2	PFIZER	29.1	NO	NO	NO	NO	NO	NO	NO	NO	NO
TN34*	38	W	Hospital	Healthy Control	NO	NO	NO	NO	YES	2	PFIZER	21.7	NO	NO	NO	NO	NO	NO	NO	NO	NO
TN36*	32	W	Hospital	Healthy Control	NO	NO	NO	NO	YES	1	PFIZER	19.0	NO	NO	NO	NO	NO	NO	NO	NO	NO
TN35*	26	W	Hospital	Healthy Control	NO	NO	NO	NO	YES	2	PFIZER	18.4	NO	NO	NO	NO	NO	NO	NO	NO	NO

Part III. Human participants characteristics of scent stimuli for Line-up procedure: test phase of Delta (new) samples**.** Controls are in green, symptomatics in orange and asymptomatics in yellow.

https://doi.org/10.3390/ani16030480

SAMPLE NUMBER	Age	Gender	Collection location	Group assigned	Cough	Anosmia	Diarrhea	Fever	Vaccination	Vaccination _ number of doses	Type of Vaccine	Body mass Index	Active smoking	Diabetes	High Blood Pressure	Dyslipidemia	Ischemic cardiopathy	Cancer	Severe renal failure	Cirhhosis	Pregnancy
	Years	Women/Men	Hospital/Home	Asymptomatic /Symptomatic / Healthy Control	YES/NO	YES/NO	YES/NO	YES/NO	YES/NO	0/1/2/3		kg/M2	YES/NO	YES/NO	YES/NO	YES/NO	YES/NO	YES/NO	YES/NO	YES/NO	YES/NO
TO1	40	M	Hospital	Asymptomatic	NO	NO	NO	NO	YES	3	PFIZER / MODERNA	21.4	NO	NO	NO	NO	NO	NO	NO	NO	NO
TO2	74	W	Home	Asymptomatic	NO	NO	NO	NO	YES	3	PFIZER	18.9	NO	NO	NO	NO	NO	NO	NO	NO	NO
TO3	59	M	Home	Asymptomatic	NO	NO	NO	NO	YES	2	MODERNA / PFIZER	25.0	NO	NO	NO	NO	NO	YES	NO	NO	NO
TO4	34	W	Home	Asymptomatic	NO	NO	NO	NO	YES	3	PFIZER	25.0	NO	NO	NO	NO	NO	NO	NO	NO	YES
TO5	30	M	Home	Asymptomatic	NO	NO	NO	NO	YES	3	ASTRAZENECA / PFIZER	30.0	NO	NO	NO	NO	NO	NO	NO	NO	NO

Part IV. Human participants characteristics of scent stimuli for Line-up procedure: test phase of Omicron AS (new) samples (in blue)**.** 5 samples of controls were randomly selected from the pool of scent presented in Part III.
